# Application of Soluplus to Improve the Flowability and Dissolution of Baicalein Phospholipid Complex

**DOI:** 10.3390/molecules22050776

**Published:** 2017-05-11

**Authors:** Junting Fan, Yunhao Dai, Hongxue Shen, Jianming Ju, Zhiying Zhao

**Affiliations:** 1 School of Pharmacy, Nanjing Medical University, Nanjing 211166, China; Juntingfan@njmu.edu.cn; 2 Affiliated Hospital of Integrated Traditional Chinese and Western Medicine, Nanjing University of Chinese, Nanjing 210028, China; 15951938737@163.com; 3 Jiangsu Province Academy of Traditional Chinese Medicine, Nanjing 210028, China; shx106@sina.com; 4 State Key Laboratory of Natural Medicines, China Pharmaceutical University, Nanjing 211198, China

**Keywords:** baicalein, phospholipid complex, soluplus

## Abstract

In this study, a novel ternary complex system (TCS) composed of baicalein, phospholipids, and Soluplus was prepared to improve the flowability and dissolution for baicalein phospholipid complex (BPC). TCS was characterized using differential scanning calorimetry (DSC), infrared spectroscopy (IR), powder X-ray diffraction (PXRD), and scanning electron microscopy (SEM). The flowability, solubility, oil–water partition coefficient, in vitro dissolution, and in vivo pharmacokinetics of the system were also evaluated. DSC, IR, PXRD, and SEM data confirmed that the crystal form of baicalein disappeared in BPC and TCS. Furthermore, the angle of repose of TCS of 35° indicated an improvement in flowability, and solubility increased by approximately eight-fold in distilled water when TCS was compared with BPC (41.00 ± 4.89 μg/mL vs. 5.00 ± 0.16 μg/mL). Approximately 91.24% of TCS was released at the end of 60 min in 0.5% SDS (pH = 6.8), which suggested that TCS could improve the dissolution velocity and extent. Moreover, TCS exhibited a considerable enhancement in bioavailability with higher peak plasma concentration (25.55 μg/mL vs. 6.05 μg/mL) and increased AUC_0–∞_ (62.47 μg·h/mL vs. 50.48 μg·h/mL) with 123.75% relative bioavailability compared with BPC. Thus, Soluplus achieved the purpose of improving the flowability and solubility of baicalein phospholipid complexes. The application of Soluplus to phospholipid complexes has great potential.

## 1. Introduction

**Figure 1:**
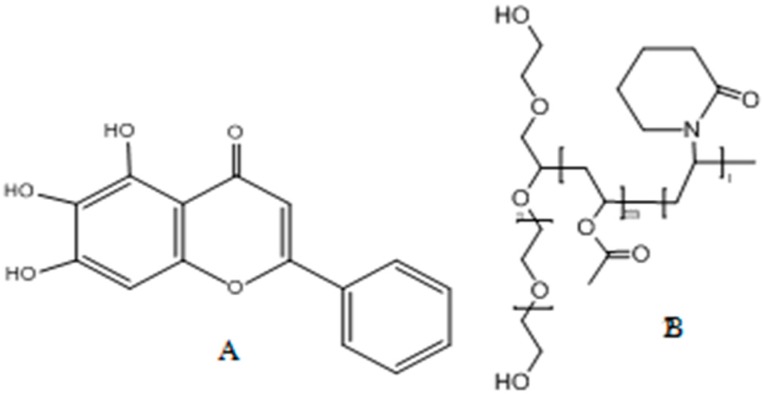
Chemical Structure of (**A**) baicalein and (**B**) Soluplus.

Baicalein (Figure 1A) is a bioactive ingredient of Radix Scutellariae. Baicalein has been reported with various pharmacological effects, such as anti-cancer [1], anti-tumor [2], anti-inflammatory [3], anti-pathogen [4], and antioxidant functions [5]. Ma’s research showed that baicalein plays a vital role in suppressing metastasis of breast cancer cells through downregulation of both SATB1 and Wnt/β-catenin [6]. Moreover, baicalein could regulate bone formation via the mTORCI pathway [7]. However, Wu reported that baicalein is a Biopharmaceuticals Classification System (BCS) class IV compound because of its low solubility (solubility of 0.052 mg/mL in water) and poor lipophilicity (Papp = 0.037 × 10^−6^ cm/s) [8]. The poor solubility and permeability of baicalein limit its oral absorption and bioavailability.

Given the phospholipid’s excellent biocompatibility and unique amphiphilicity, a drug–phospholipid complex has been employed as a technique to improve the oral absorption of the drugs which belong to BCS class III and IV [9,10,11]. Devendra Singh Rawat pointed out that the water/*n*-octanol solubility of baicalein was improved in baicalein phospholipid complex (BPC) [12]. Nevertheless, the drug–phospholipid complex’s strong lipid solubility can have a disadvantageous influence on the drug dissolution rate. Moreover, the non-flowing character of BPC as a semi-solid station limits its application in solid preparation.

Soluplus (Figure 1B) has been used as an excipient in many reports and with other processing methods, such as spray drying [13,14,15,16]. Soluplus has a natural amphiphilic structure that makes it miscible with hydrophobic drugs and maintains its aqueous solubility because of vinyl acetate, vinyl caprolactam blocks, and ethylene glycol blocks [17,18]. Hua Yang applied Soluplus as a stabilizer in the preparation of a nanosuspension to improve the bioavailability of fenofibrate [19]. Alireza Homayouni adopted antisolvent precipitation and high-pressure homogenization techniques with Soluplus to enhance the dissolution of Celecoxib [20]. Andres Lust prepared piroxicam and Soluplus into amorphous solid dispersions for qualitative and quantitative analyses of recrystallization during storage [21,22].

In the present study, a novel ternary complex system (TCS) composed of baicalein, phospholipids, and Soluplus was prepared to improve the flowability and dissolution in vitro of BPC. Due to ameliorative flowability, the successful development of TCS has created favorable conditions for large-scale production of baicalein in industrial production. Likewise, the improvement in dissolution is also advantageous for the improvement of bioavailability. Based on these findings, we conclude that Soluplus achieved the purpose of improving the flowability and solubility of BPC. The safe and effective excipients incorporation of phospholipid complex provides a new idea for its better development. In our design, TCS was prepared via solvent evaporation with baicalein, phospholipids, and Soluplus. First, TCS was characterized using differential scanning calorimetry (DSC), infrared spectroscopy (IR), powder X-ray diffraction (PXRD), and scanning electron microscopy (SEM). Second, flowability, solubility, oil–water partition coefficient, and dissolution in vitro were evaluated. Finally, the baicalin content was detected in rats after oral administration to further assess bioavailability via High Performance Liquid Chromatography-Electrospray Ionization-Mass Spectrometry/Mass Spectrometry (HPLC-ESI-MS/MS) [23].

## 2. Materials and Methods

### 2.1. Materials

Baicalein and baicalin were purchased from Sichuan Weikeqi Biology Technique Co., Ltd. Soluplus was kindly gifted by BASF SE (Ludwigshafen, Germany). Icariin was purchased from the China Institute of Pharmaceutical and Biological Products and employed as an internal standard. All other chemical reagents used in the experiments were analytical grade or better. Purified Milli-Q water was utilized in the experiments (Millipore, Billerica, MA, USA).

### 2.2. Methods

#### 2.2.1. Preparation of Samples

##### Preparation of BPC

The drug and phospholipid (1:2 mass ratio) were used to prepare BPC. In brief, baicalein and phospholipid (phosphatidylcholine) were dissolved in 10 mL of absolute ethanol. The solution was magnetically stirred for 0.5 h and then vacuum dried for 24 h to obtain the solid product, which was stored in a desiccator.

##### Preparation of TCS

The drug and phospholipid (BPC) with Soluplus were weighed at a mass ratio of 1:2:2 and dissolved in 10 mL of absolute ethanol. The first two were mixed and dissolved by magnetic stirring for 0.5 h. Then, the weighed Soluplus was added to the above mixed solution, stirring to dissolve completely. Further stirring for 0.5 h resulted in a Soluplus complex with mass ratios of 1:2:2. The complex was vacuum dried for 24 h to obtain the solid powder, which was then stored in a desiccator.

##### Preparation of Physical Mixture (PM) of TCS

PM was prepared by thoroughly mixing baicalein, phospholipid, and Soluplus (1:2:2 mass ratio) in a mortar for 10 min until a homogenous mixture was obtained. The sample was stored in a desiccator.

#### 2.2.2. Characterization of the Sample

##### Differential Scanning Calorimetry (DSC)

The thermal properties of Baicalein, BPC, TCS, and PM of TCS were studied using a differential scanning calorimeter (DSC 449F3, Netzsch, Selb, Germany) equipped with a thermal analysis system. Dry nitrogen was used as the purge gas (purge 40 mL/min). The right amount of samples of baicalein, BPC, TCS, and PM were weighed into an aluminum pan. The probes were heated at a temperature of 0–300 °C at a rate of 20 °C/min [24].

##### Infrared Spectroscopy (IR)

The IR spectra of baicalein, BPC, TCS, and PM of TCS were obtained using an IR spectrophotometer (Ominic, New York, NY, USA). Samples were scanned over the wavenumber range of 4000–400 cm^−1^.

##### Powder X-ray Diffraction (PXRD)

The crystalline state of prepared baicalein, BPC, TCS, and PM of TCS was acquired at room temperature with Cu Kα radiation source at 40 kV and 25 mA via XRD (Bruker AXS, D8, Karlsruhe, Germany).

##### Scanning Electron Microscopy (SEM)

The granule morphology of baicalein, BPC, TCS, and PM of TCS were determined using a SEM (6390LV, Tokyo, Japan). The samples were sputter-coated (E-1010, Hitachi Ltd., Tokyo, Japan) with gold–palladium and then observed at different magnifications.

#### 2.2.3. Flowability

After TCS was added into the hopper, it was allowed to flow uniformly through the funnel until the highest point of the cone. The height (h) and radius (r) of the cone were then measured to calculate the angle of repose (θ).

#### 2.2.4. Solubility

Water or *n*-octanol (5.0 mL) was added to excess baicalein, BPC, and TCS to determine solubility in the swing bed at 37 °C for 24 h [25]. The liquids were then shaken to balance and centrifuged at 15,000 rpm for 10 min to remove excess baicalein and TCS. A 10 μL aliquot of the resulting solution was injected into the HPLC system before the supernatant was filtered using a 0.45 μm membrane. Experiments were performed in triplicate.

#### 2.2.5. Oil–Water Partition Coefficient Studies

After 2.0 mL of baicalein, BPC, or TCS-saturated *n*-octanol (water saturation) was shaken to balance, 2.0 mL of water (*n*-octanol saturation) was added. The miscible liquid was then agitated for 24 h, and the concentration in each phase was determined using HPLC after standing for layering. Experiments for each sample were performed in triplicate.

#### 2.2.6. In Vitro Dissolution Studies

##### Chromatographic Conditions

All samples were analyzed using Waters HPLC with a quaternary pump (Waters 2695 separation module, Waters 2996 PDA detector) on a Diamonsil C18 column (200 mm × 4.6 mm × 5 μm) at 276 nm, which was maintained at 35 °C. The mobile phase was methanol-0.1% formic acid (60:40, *v/v*), and the flow rate was 1.0 mL/min. The injection volume was 10 μL. The method was optimized from the protocol described by Zhang [26].

##### Dissolution

The rotating basket method with automated dissolution apparatus (RCZ-8M, Shanghai, China) was used according to appendix XC of Chinese Pharmacopoeia 2010 edition. The samples, which were equivalent to 10 mg of baicalein, were filled in hard gelatin capsules and placed in the dissolution vessel containing 900 mL of 0.5% SDS with phosphate buffer (pH 2.0 or 6.8). The vessel was maintained at 37 °C ± 0.5 °C and stirred at 100 rpm. Approximately 2.0 mL of sample was withdrawn from the dissolution medium and replaced by an equivalent volume of fresh medium at 5, 10, 20, 30, 45, and 60 min. The baicalein content was determined using HPLC after the sample was filtered through a 0.45 μm membrane. Experiments for each sample were performed in triplicate.

#### 2.2.7. Pharmacokinetic Study In Vivo

##### Animals

A total of 24 male SD rats (SPF grade) weighing 220 ± 10 g were purchased from Benbu Yinuogui Biology Technique Ltd. The rats were kept in the Animal Research Center to acclimatize to a new environment. The rats were then randomly divided into four groups—namely, baicalein, BPC, TCS, and PM of TCS.

##### Plasma Sample Preparations

All animal experimental protocols were approved by the Animal Care Committee of the Institute of Chinese Medicine in Jiangsu Province. The rats were fasted for 12 h and provided free access to water prior to the experiment. Each group was orally administered with a single dose equivalent to 40 mg/kg baicalein [27]. An aliquot of 300 μL of blood samples was taken from the eye ground veins at 0.12, 0.25, 0.50, 0.75, 1.0, 1.5, 2.0, 3.0, 5.0, 9.0, 13, and 24 h after oral administration. The supernatant was retained after centrifugation at 4500 rpm for 10 min. Plasma samples were collected and stored at −80 °C until further use.

##### Plasma Sample Handing

Baicalein, baicalin (corresponding to 5.75 and 5.06 μg/mL), 100 μL of plasma, 100 μL of mixed standard solution, and 10 μL of internal standard solution (5.60 μg/mL) were mixed and vortexed for 15 s. Approximately 0.5 mL of methanol and 100 μL of 1 M KH_2_PO_4_ were added and vortexed for another 3 min. The mixture was centrifuged at 15,000 rpm for 5 min prior to evaporation under nitrogen. The dried residue was dissolved in 100 μL of methanol–distilled water (1:1, *v/v*). After being vortexed at 15,000 rpm for 5 min, the supernatant was injected for HPLC-ESI-MS/MS analysis. The MS/MS system (Waters-2695-MicroMass Quattro Micro, Milford, CT, USA) was operated under positive mode and multiple reaction monitoring mode. The MS conditions were as follows: ion spray voltage of +5.5 kV; nitrogen as nebulizer gas, auxiliary gas, and curtain gas at 30, 60, and 10 psi, respectively; collision gas set at medium; and auxiliary gas temperature of 400 °C.

##### Analysis of Date 

The maximum concentration (C_max_) and maximum time (T_max_) were directly determined from the concentration–time data. Other pharmaceutical parameters (AUC_0–24 h_ and t_1/2_) were analyzed using pharmacokinetic program DAS 2.1.1. ANOVA was performed using SPSS 16.0 software.

## 3. Results and Discussion

### 3.1. Particle Morphology

**Figure 2:**
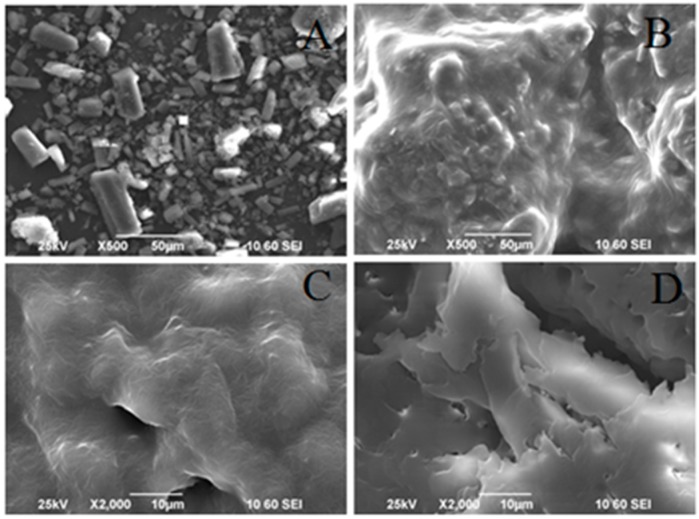
SEM diagrams of (**A**) baicalein; (**B**) physical mixture (PM) of ternary complex system (TCS, 1:2:2); (**C**) baicalein phospholipid complex (BPC, 1:2); and (**D**) TCS (1:2:2).

The SEM images of baicalein, PM of TCS, BPC, and TCS are presented in Figure 2. Baicalein (Figure 2A) exhibited an almost rectangular crystal. TCS (Figure 2D) was flake-like in shape, which was a great change compared with baicalein, PM of TCS (Figure 2B), and BPC (Figure 2C). The disappearance of crystals in TCS indicated the complete miscibility of the drug, phospholipid, and Soluplus. PM of TCS also exhibited crystals, although they were wrapped by phospholipid and Soluplus.

### 3.2. Infrared Spectroscopy

**Figure 3:**
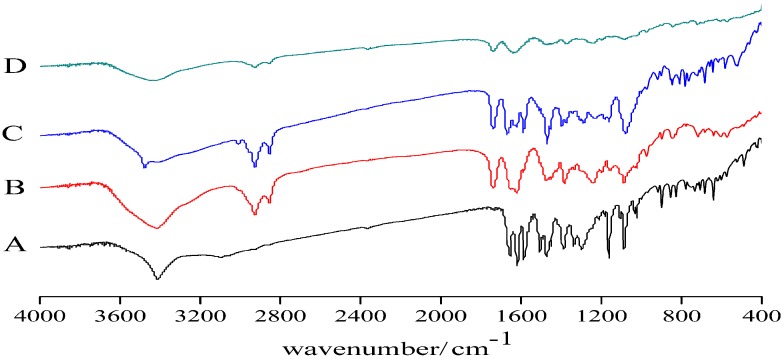
Infrared spectroscopy (IR) diagrams of baicalein (**A**); PM of TCS (1:2:2) (**B**); BPC (1:2) (**C**); and TCS (1:2:2) (**D**).

Figure 3 shows the IR spectra of the samples, which confirmed the formation of TCS compared with the original drug based on the characteristic chemical bond. PM of TCS (Figure 3B) exhibited a hydroxyl stretching band at 3478.68 cm^−1^ and carbon hydrogen bond at 2919 cm^−1^. The spectrum of baicalein phospholipid complex also had the characteristics of baicalein infrared spectra, which was consistent with the trend of the infrared spectrum of PM, and there was no significant difference, indicating that the formation of phospholipid complex did not produce new chemical bonds between molecules. However, the intensity of both chemical bonds in TCS (Figure 3D) was weaker. The overall trend of the curve and the characteristic peaks had undergone great changes because of the presence of the Soluplus in phospholipid complex—all results indicated TCS formation.

### 3.3. X-ray Diffraction Pattern

**Figure 4:**
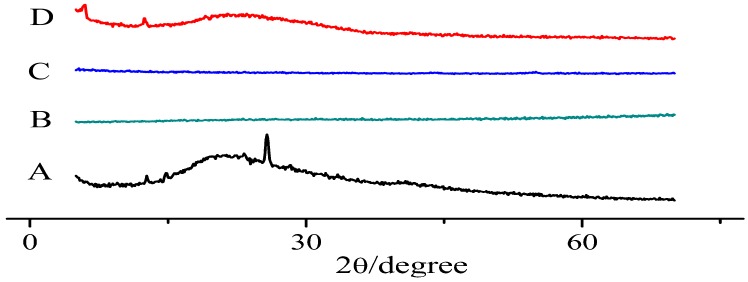
X-ray diffraction spectra of baicalein (**A**); TCS (1:2:2) (**B**); BPC (1:2) (**C**); and PM (1:2:2) (**D**).

The X-ray diffraction patterns of baicalein, PM of TCS, TCS, and BPC are shown in Figure 4. The diffraction peaks of baicalein crystal were observed at a diffraction angle of 2θ, indicating that the drug was present as a crystalline material. Characteristic baicalein peaks also appeared in PM, but disappeared in TCS and BPC. This result suggested that baicalein in TCS completely existed in the amorphous phase.

### 3.4. Differential Scanning Calorimetry

**Figure 5:**
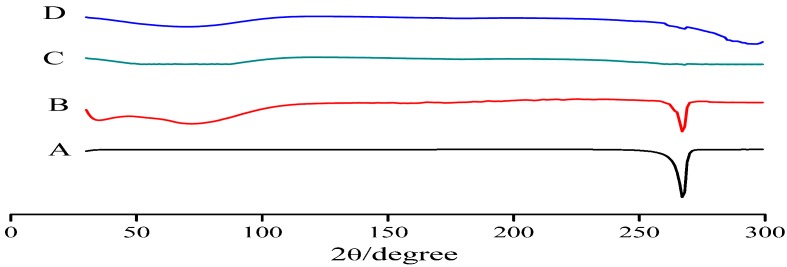
Differential scanning calorimetry (DSC) spectra of baicalein (**A**); PM (1:2:2) (**B**); BPC (1:2) (**C**); and TCS (1:2:2) (**D**).

DSC can screen drug–excipient compatibility and provide information about the interactions between them. Figure 5 shows the DSC spectra of baicalein (Figure 5A), PM of TCS (Figure 5B), BPC (Figure 5C), and TCS (Figure 5D). DSC thermograms showed that baicalein (Figure 5A) had an endothermic peak at about 265 °C corresponding to the melting point of baicalein which suggested baicalein crystal formation. The weaker peak appearing at the same temperature in Figure 5B indicates that baicalein still exists in the form of crystallization in PM, whereas Figure 5C,D showed a horizontal line. We speculated that the melting point of a drug phospholipid complex may be changed so as to make it undetectable using DSC. These observations indicated that baicalein in TCS could exist in amorphous form due to the possible inhibitory effect of Soluplus on drug crystallization, which was consistent with the SEM results.

### 3.5. Flowability of TCS

The flow of powders as assessed by the angle of repose is based on the inter-particle cohesion: Values less than 25° is suggestive of “very-good flow,” whereas values equal to and greater than 25° but less than 50° show “good flow”, and values greater than 50° indicate “poor flow” [28]. The flowability of TCS was 35° according to the formula: tan θ = h/r, qualified as having “good flow”. By contrast, the flowability of the BPC could not be measured due to the semi-solid state of the BPC, which was defined as a non-flowing material. Soluplus as a carrier played a role in the curing of BPC; TCS had a significant improvement in flowability compared with BPC, thus achieving the purpose of this experiment.

### 3.6. Solubility and Oil–Water Partition Coefficient

**Figure 6:**
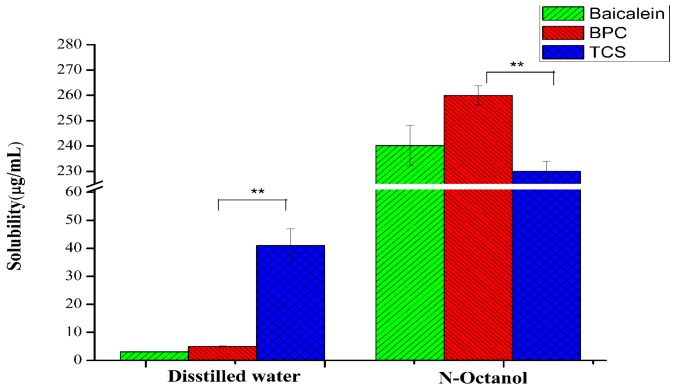
Solubility of baicalein and TCS in distilled water and *n*-octanol. Values are mean ± SD (*n* = 3). ** *p* < 0.01.

Figure 6 displays the solubility data of baicalein, BPC, and TCS in distilled water and *n*-octanol. Table 1 exhibits the log *p* values of baicalein, BPC, and TCS. The solubility of TCS (41 ± 4.89 μg/mL) in distilled water was higher than that of BPC (5.02 ± 0.09 μg/mL) (*p* < 0.01). The solubility of TCS (230 ± 8.78 μg/mL) in *n*-octanol was slightly lower than that of BPC (260 ± 7.52 μg/mL). Moreover, TCS had lower log *p* values than BPC (2.01 vs. 2.04). The increase in solubility in distilled water and decrease in *n*-octanol may be caused by the natural hydrophilic structure of Soluplus. Therefore, TCS could enhance the water solubility of BPC.

### 3.7. Dissolution Study

**Figure 7:**
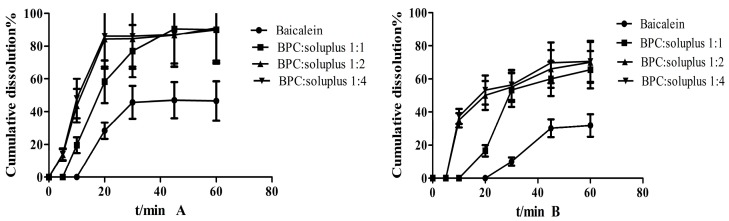
Different proportions (*m*/*m*) of BPC and Soluplus in dissolution experiment in 0.5% SDS with (**A**) phosphate buffer (pH = 6.8) and (**B**) phosphate buffer (pH = 2.0).

Figure 7 shows the cumulative dissolution of different proportions between BPC (the mass proportion of baicalein and phospholipid was 1:2) and Soluplus. Baicalein dissolved to almost 40% and 30% in 60 min in 0.5% SDS with phosphate buffer at pH 6.8 and pH 2.0, respectively. TCS exhibited nearly 90% better dissolution extent relative to baicalein. When the mass ratio of BPC and Soluplus was 1:2, the amount of dissolution was higher than that of the ratio at 1:1; however, no distinguishable enhancement in dissolution was exhibited at 1:4 compared with 1:2. Two dissolution media showed the same phenomenon. Based on the present results, we can draw the conclusion that the optimal mass ratio of BPC and Soluplus is 1:2.

**Figure 8:**
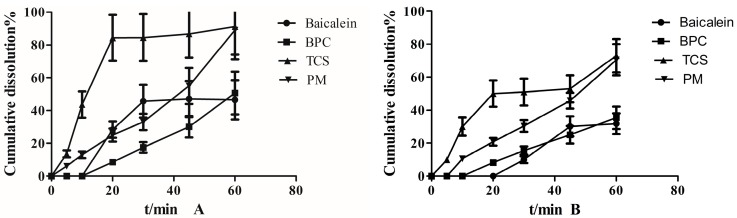
Dissolution behavior of baicalein, BPC, TCS, and PM of TCS in 0.5% SDS with (**A**) phosphate buffer (pH = 6.8) and (**B**) phosphate buffer (pH = 2.0).

Figure 8 presents the dissolution profiles of baicalein, BPC, TCS, and PM of TCS in 0.5% SDS with phosphate buffer at pH 6.8 and pH 2.0. TCS exhibited higher dissolution with respect to BPC at the end of 60 min in A and B (at 91.24% vs. 46.58% and 73.35% vs. 35.43%, respectively). Moreover, TCS increased to 84.26% in 20 min, which was nine-fold higher than BPC (8.55%) in A, and increased to 50.56% in B compared with BPC (8.30%). Two groups of BPC and baicalein showed no dissolution after 10 min, which indicated that the viscosity of the phospholipid complex could hinder the dissolution velocity, and the phospholipid decreased baicalein’s water solubility.

### 3.8. Bioavailability Analysis

**Figure 9:**
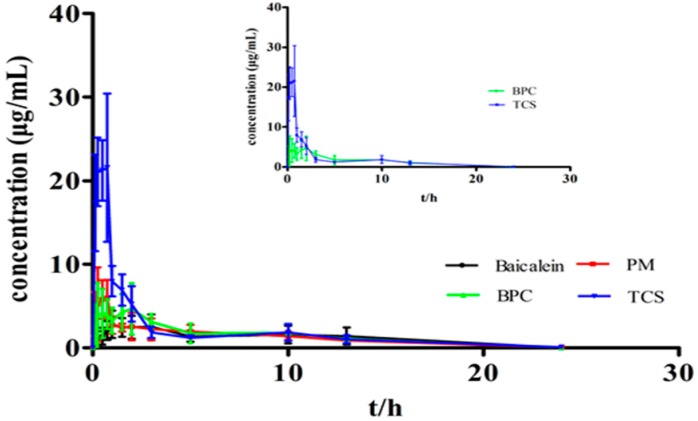
Mean plasma concentration–time curves of baicalin after oral administration of baicalein, BPC, TCS, and PM of TCS (data are represented as mean ± SD, *n* = 6).

Plasma concentration–time profiles are presented in Figure 9, and the corresponding pharmacokinetic parameters are summarized in Table 2. The present study showed only one peak, which was in accordance with the findings of previous studies [29], although other studies reported a two-peak phenomenon [30,31]. Given that the content of baicalein cannot be detected after oral administration, the baicalin is predominant in the plasma when baicalein is administered orally, so baicalein absorption can be assessed by detecting the baicalin concentration and baicalein glycosides. TCS peaked at 0.63 h (25.55 μg/mL), showing considerable improvement (*p* < 0.01) compared with BPC, which peaked at 1.01 h (6.05 μg/mL). TCS exhibited a marked enhancement compared with BPC in oral bioavailability, with an increase in AUC_0–24 h_ (53.16 μg·h/mL vs. 38.40 μg·h/mL) (*p* < 0.05), and AUC_0–∞_ (62.47 μg·h/mL vs. 50.48 μg·h/mL) (*p* < 0.05). The relative bioavailability of TCS was approximately 123.75% compared with BPC, confirming the enhanced bioavailability in the complex. Similarly, a four-fold increase in C_max_ (25.55 μg/mL vs. 6.05 μg/mL) was observed.

Soluplus was adopted as a hydrophilic pharmaceutical excipient to improve solubility, in vitro dissolution, or in vivo bioavailability in previous studies, such as solid dispersion [16], nanosuspension [19], and self-emulsification [32]. Soluplus was also used as a stabilizer to prevent agglomeration and crystal growth by reducing the surface energy of fine particles [33]. In the present study, a considerable enhancement was observed in the dissolution rate and extent in vitro and flowability of BPC by means of the application of Soluplus. The in vivo pharmacokinetic study showed that TCS could improve C_max_ and AUC_0–∞_ of BPC. All these results demonstrated that TCS may be applied to baicalein’s oral solid preparation.

## 4. Conclusions

In our study, a novel TCS composed of baicalein, phospholipids, and Soluplus was successfully developed. The 35° angle of repose of TCS indicated an improvement in flowability, which met the industrial demand (θ < 40°). Moreover, TCS exhibited a marked enhancement in both the rate and extent of dissolution in vitro, as well as the bioavailability parameters C_max_ and AUC_0–24 h_, compared with BPC. The preparation method is simple and convenient, but also for Soluplus as a safe and effective drug excipient to explore a new pharmaceutical application. In conclusion, TCS is a promising method to improve the flowability and dissolution for drug–phospholipid complex.

## Figures and Tables

**Table 1:** Log *p* values of baicalein, BPC and TCS.

Sample	Baicalein Concentration in Aqueous Phase Cw (μg/mL)	Baicalein Concentration in Organic Phase Co (μg/mL)	Partition Coefficient (Co/Cw)	Log *p* (Co/Cw)
Baicalein	0.17 ± 0.03	2.01 ± 0.05	11.82	1.07
BPC	0.13 ± 0.02	25.08 ± 1.02	109.04	2.04
TCS	0.25 ± 0.01	26.01 ± 0.95	104.04	2.01

**Table 2:** Pharmacokinetic parameters of baicalein, BPC, TCS, and PM of TCS after single oral administration of 40 mg/kg body weight to rats (*n* = 6).

Parameters	Baicalein	PM	BPC	TCS
C_max_ (μg/mL)	2.87 ± 1.82	8.67 ± 2.04	6.05 ± 3.02	25.55 ± 1.11 **
T_max_ (h)	1.44 ± 1.05	0.75 ± 0.26	1.01 ± 0.74	0.63 ± 0.25
AUC_0–24 h_ (μg·h/mL)	24.93 ± 13.13	30.01 ± 8.44	38.40 ± 10.32	53.16 ± 6.71 *
AUC_0–∞_ (μg·h/mL)	40.99 ± 13.35	43.25 ± 8.50	50.48 ± 10.34	62.47 ± 7.11 *
t_1/2_ (h)	2.32 ± 0.33	3.05 ± 0.77	2.24 ± 0.22	3.09 ± 0.65
MRT (0–∞)	7.43 ± 1.04	6.48 ± 0.49	6.80 ± 0.97	5.04 ± 1.11

MRT (0–∞): mean residence time ** *p* < 0.01, * *p* < 0.05 compared with BPC.
